# Genomic insights into an obligate epibiotic bacterial predator: *Micavibrio aeruginosavorus *ARL-13

**DOI:** 10.1186/1471-2164-12-453

**Published:** 2011-09-21

**Authors:** Zhang Wang, Daniel E Kadouri, Martin Wu

**Affiliations:** 1 Department of Biology, University of Virginia, 485 McCormick Road, Charlottesville, Virginia 22903, USA; 2 Department of Oral Biology, University of Medicine and Dentistry of New Jersey, Newark, New Jersey 07101, USA

**Keywords:** Bacterial predation, Predator-prey interaction, Integrative and conjugative elements (ICEs), Hemolysin-related protein, Quorum sensing, RNA-Seq

## Abstract

**Background:**

Although bacterial predators play important roles in the dynamics of natural microbial communities, little is known about the molecular mechanism of bacterial predation and the evolution of diverse predatory lifestyles.

**Results:**

We determined the complete genome sequence of *Micavibrio aeruginosavorus *ARL-13, an obligate bacterial predator that feeds by "leeching" externally to its prey. Despite being an obligate predator depending on prey for replication, *M. aeruginosavorus *encodes almost all major metabolic pathways. However, our genome analysis suggests that there are multiple amino acids that it can neither make nor import directly from the environment, thus providing a simple explanation for its strict dependence on prey. Remarkably, despite apparent genome reduction, there is a massive expansion of genomic islands of foreign origin. At least nine genomic islands encode many genes that are likely important for *Micavibrio*-prey interaction such as hemolysin-related proteins. RNA-Seq analysis shows substantial transcriptome differences between the attack phase, when *M. aeruginosavorus *seeks its prey, and the attachment phase, when it feeds and multiplies. Housekeeping genes as well as genes involved in protein secretion were all dramatically up-regulated in the attachment phase. In contrast, genes involved in chemotaxis and flagellum biosynthesis were highly expressed in the attack phase but were shut down in the attachment phase. Our transcriptomic analysis identified additional genes likely important in *Micavibrio *predation, including porins, pilins and many hypothetical genes.

**Conclusions:**

The findings from our phylogenomic and transcriptomic analyses shed new light on the biology and evolution of the epibiotic predatory lifestyle of *M. aeruginosavorus*. The analysis reported here and the availability of the complete genome sequence should catalyze future studies of this organism.

## Background

Predatory bacteria are a diverse group of bacteria that attack and feed on other bacteria. They live in various habitats and likely play an important role in microbial ecosystems [1-3]. Predation probably has originated multiple times in Bacteria, as examples of predators have been found in dispersed major lineages including *Proteobacteria*, *Chloroflexi*, *Cytophagaceae*, and Gram-positive bacteria [4]. Bacterial predators prey using a number of strategies. For example, *Myxobacteria *are facultative predators. They attack as a "wolf pack" and feed on, among other substrates, various live and dead bacteria. On the other hand, *Bdellovibrio *and like organisms (BALOs) are obligate predatory bacteria -- they can only survive by preying on other bacteria [5]. Unlike *Myxobacteria*, which use excreted hydrolytic enzymes to degrade prey cells, obligate predation requires close and irreversible contact between the predator and the prey. *Bdellovibrio *invade the periplasmic space of their prey, where they replicate at the expense of the prey's cellular content and eventually lyse the cell. *Micavibrio*, on the other hand, feed by "leeching" externally to the surface of the prey cell and therefore has an epibiotic lifestyle [6-9].

First isolated in 1983 from wastewater, *Micavibrio aeruginosavorus *is Gram-negative, relatively small in size (0.5 to 1.5 μm long), rod shaped, curved and has a single polar flagellum [7]. Like BALOs, *Micavibrio spp*. are characterized by an obligatory parasitic life cycle. *Micavibrio's *life cycle is believed to consist of an attack phase, in which motile *Micavibrio *seek their prey, and an attachment phase, in which *Micavibrio *attach irreversibly to the cell surfaces of prey bacteria. At this point the attached *Micavibrio *feed on their prey and divide by binary fission, leading to the death of the infected prey cells [7,9-11]. *Micavibrio *usually exhibit a high degree of prey specificity. For example, *M. aeruginosavorus *was initially reported to prey only on *Pseudomonas aeruginosa*, *Burkholderia cepacia *and *Klebsiella pneumoniae *[7,8]. However a breach in prey specificity was recently demonstrated and *M. aeruginosavorus *was found to be able to prey on many other bacterial species including *Escherichia coli *[6].

*Myxobacteria *and *Bdellovibrio*, both belonging to the delta-proteobacteria, have been extensively studied [12,13]. Members from both groups (*M. xanthus *DK1622 and *B. bacteriovorus *HD100) have recently been sequenced [14,15]. In comparison, *Micavibrio*, members of the alpha-proteobacteria, have received much less attention, at least partly due to the difficulty to obtain axenic culture and partly due to the lack of good genetic tools to study them. In order to gain greater insights into its predatory lifestyle and to further understand the evolution of bacterial predation in general, we sequenced one of the better studied strains, *Micavibrio aeruginosavorus *ARL-13 [6,8,9] and characterized its transcriptome during the attachment and attack stages of its growth cycle.

## Results and Discussion

### Genome summary

**Figure 1:**
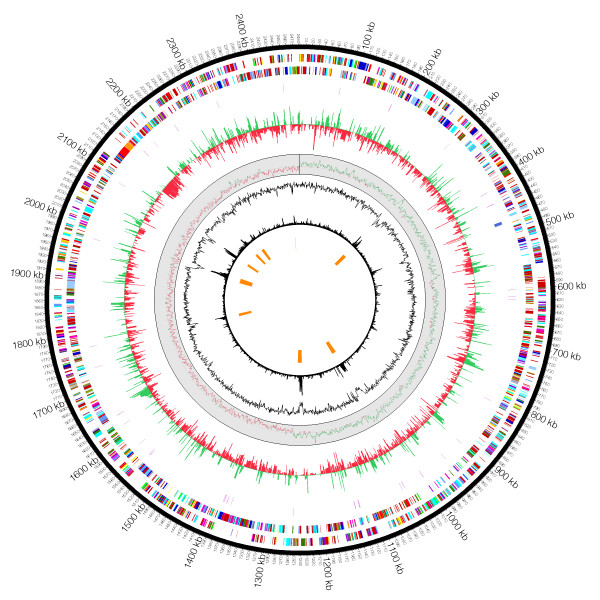
**Main features of the *M. aeruginosavorus *chromosome**. From the outside inward the circles show: (1) and (2) predicted protein-coding regions on the plus and minus strands (colors were assigned according to the color code of functional classes; (3) tRNA genes (purple) and rRNA genes (blue); (4) gene expression level as measured by the natural logarithm of Gene Expression Index (GEI), attack phase (green) and attachment phase (red); (5) GC skew plot; (6) GC%; (7) tri-nucleotide chi-square score; (8) genomic islands.

The complete genome of *Micavibrio aeruginosavorus *ARL-13 consists of 2,481,983 base pairs on a single circular molecule with a G+C content of 54.7%. Major features of the genome are summarized in Table 1 and Figure 1. The genome exhibits two clear GC skew transitions that likely correspond to the DNA replication origin and terminus (Figure 1). 90.3% of the genome is predicted to code for 2434 open reading frames (ORFs), 40 tRNA genes and one rRNA operon. Only 50.5% of the predicted ORFs can be assigned to a putative function. No extragenomic DNA molecules (plasmid or phage) were identified from the genome sequence assembly. CRISPRs (**C**lustered **R**egularly **I**nterspaced **S**hort **P**alindromic **R**epeats) function as the immune system of bacteria and archaea that defends against exogenous DNA such as phages and plasmids [16]. Accordingly, no CRISPRs elements were identified from the genome.

**Table 1:** Main features of the genome of *M.aeruginosavorus *ARL-13

Feature	Value
Genome Size, bp	2,481,983
GC%	54.7
Predicted open reading frames (ORFs)	2434
ORFs with assigned function	1228 (50.5%)
Conserved hypothetical ORF	193 (7.9%)
Unknown function ORF	124 (5.1%)
Hypothetical ORF	746 (30.6%)
Average ORF length, bp	919
Percent of genome that is coding	90.3
Ribosomal RNA operon	3
Transfer RNA	40
CRISPR element	0
Plasmid	0

Repetitive DNAs facilitate genome arrangement and increase the genome plasticity through homologous recombination. Strikingly, only 0.10% of the *M. aeruginosavorus *genome is repetitive (at least 50 bp with at least 97% identity; in comparison, 2.7% of *E. coli *genome contains repeats). The only large repeat (> 100 bp) that can be identified from the genome is a 1200 bp fragment encoding the elongation factor Tu gene, whose duplication is known to be widespread among proteobacteria [17]. The genome is completely devoid of mobile genetic elements including transposons, retrotransposons and insertion sequences. The paucity of repetitive DNA has been attributed to extensive genome streamlining [18]. Observations of genomes with such an infrequent occurrence of repeats have been limited to obligate intracellular bacteria (e.g., *Buchnera*, *Rickettisa *and *Chlamydiales*) and the free-living bacteria *Prochlorococcus *and *Pelagibacter *that have gone through extensive genome reduction [18-22]. *Micavibrio*'s genome is moderate in size. At 2.4 Mbp, it is almost twice as large as most obligate intracellular alpha-proteobacteria, but is still substantially smaller than most free-living alpha-proteobacteria, and about 35% smaller than *B. bacteriovorus *HD100 (3.7 Mbp) [14]. *M. aeruginosavorus' *genome does not have the extreme GC% bias typical of intracellular bacteria and is almost completely devoid of pseudogenes.

### Phylogeny and taxonomy

**Figure 2:**
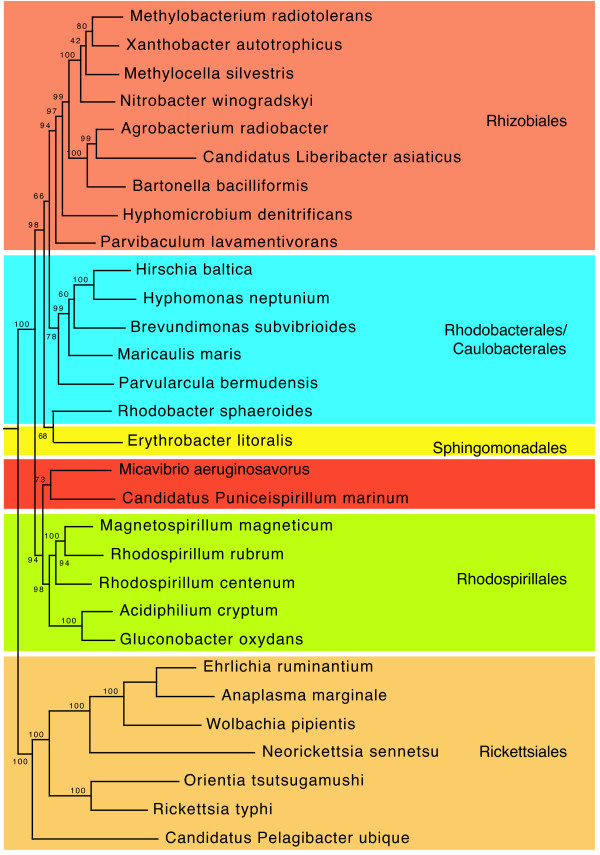
**A maximum likelihood genome tree of alpha-proteobacterial representatives**. A maximum likelihood tree was built from concatenated protein sequences of 31 universal housekeeping genes and rooted by gamma- and beta-proteobacteria. Bootstrap support values (out of 100 runs) for branches of interest are shown beside them.

*Micavibrio spp*. have many morphological and physiological features resembling those of the *Bdellovibrio spp*. As a result, historically, *Micavibrio spp*. have been affiliated with *Bdellovibrio *and classified as delta-proteobacteria [23]. However, recent studies based on the 16s rRNA and several protein-coding genes have placed *Micavibrio *as a deep branch lineage within the alpha-proteobacteria [9], which is strongly supported by our genome-level phylogenetic analysis using 31 housekeeping genes (Figure 2). Its closest relative with a sequenced genome is "*Candidatus Puniceispirillum marinum"*, a member of the ubiquitous marine bacterioplankton SAR116 group [24]. Together, they form a sister clade to the *Rhodospirillales *order that is otherwise distinct from all the major alpha-proteobacterial groups that are currently recognized. Based on our own and previous phylogenetic analyses, we recommend that the taxonomy of *Micavibrio *to be revised.

### General metabolic features

Although an obligate predator depending on prey for cell replication, *M. aeruginosavorus *has a free-living attack phase during which it swims around and seeks out the prey. Analysis of the genome shows that it has many features of a free-living bacterium (Additional file 1). For example, it has an elaborate suite of genes involved in cell wall and lipopolysaccharide (LPS) biosynthesis; it is predicted to cover all major metabolic pathways, including glycolysis, the tricarboxylic acid (TCA) cycle, the electron transport and respiration systems and ATP synthase, indicating that it is fully capable of generating ATP on its own by converting carbohydrate, fats and proteins into carbon dioxide and water. It also possesses a complete pentose phosphate pathway and a full set of genes for nucleotide metabolism, allowing it to synthesize nucleotides from scratch. Not surprisingly, it does not encode any known nucleotide transporters. It has a slightly reduced set of 43 genes devoted to biosynthesis of cofactor, prosthetic groups and carriers. Obligate intracellular bacteria such as *Buchnera *depend on their hosts for most of their nutrients, and as a result of the reduced selection pressure, they have lost a lot of biosynthetic genes [19]. The gene loss in *M. aeruginosavorus *is modest in comparison, suggesting that there is considerable selective pressure acting on the remaining genes. This is consistent with the finding that there are rarely any pseudogenes or signs of active gene degradation in the genome.

### Amino acid biosynthesis and transport

Since *M. aeruginosavorus *is an obligate predator and has not been cultured axenically, it is of particular interest to use the genome sequence to understand its nutritional needs. Analysis of the genome sequence revealed that *M. aeruginosavorus *encodes genes to synthesize 13 amino acids needed for protein synthesis. However, it is missing almost the entire biosynthesis pathways for the other 7 amino acids: Alanine, Arginine, Histidine, Isoleucine, Methionine, Tryptophan and Valine, suggesting that it can not synthesize these amino acids either de novo or from metabolic intermediates, and has to obtain them directly from external sources. Strikingly, the genome is completely devoid of any known transporters for amino acids, peptides and amines, although it contains 82 ORFs predicted to transport ions, carbohydrates, organic alcohols and acids and other unknown substrates.

Our genome analysis suggested that *M. aeruginosavorus *is deficient in amino acid biosynthesis and uptake from the environment, which at least partially explains why *M. aeruginosavorus *could not be cultured in nutrient rich media [7,9] (Daniel Kadouri, unpublished data). It would be extremely difficult for *Micavibrio *to revert to a lifestyle independent of prey, as it would entail the acquisition of many eliminated genes including those involved in amino acid metabolism. This could explain the failure to isolate prey-independent variants of *Micavibrio *using rich media as described for *Bdellovibrio *[25,26] (Daniel Kadouri, unpublished data). In contrast, although *B. bacteriovorus *is capable of synthesizing only 11 amino acids [14], it has a large repertoire of 113 transporters for transporting amino acids, peptides or amines. Therefore, *Bdellovibrio *is capable of importing amino acids that it cannot make on its own from the environment. Accordingly, spontaneous mutants of *Bdellovibrio *that grow in rich media have been isolated at a frequency of 10^-6 ^to 10^-7 ^and higher [25,27].

Among all bacterial and archaeal species sequenced to date, only a few species such as *Buchnera spp*. and *Nanoarchaeum equitans *encode no known amino acid transporters in their genomes. *Buchnera *are bacterial endosymbionts engaged in a classical example of metabolic symbiosis with their host aphids: *Buchnera *supply aphids with essential amino acids and in return, aphids provide complementary non-essential amino acids to the bacteria. The shuttling of the amino acids between the host and the endosymbiont is most likely carried out by transporters encoded by the host genome but not the bacterial genome itself [19,28]. *Nanoarchaeum equitans *represents a more interesting analogy to *Micavibrio spp*. It is an obligate epibiotic parasite that lives on another archaeon *Ignicoccus*. It attaches to the surface of the host cell and presumably acquires its nutrients from the host cell because its tiny genome of 0.5 Mbp does not encode genes for biosynthesis of amino acids, nucleotides or cofactors, nor does it encode transporters for these substrates that allow direct import from the environment [29]. Consequently, *Nanoarchaeum *must stay in direct contact with the host organism to survive.

Recently, it has been shown that bacteria can exchange cellular constituents (small molecules, proteins and DNAs) through intercellular nanotubes that connect neighboring cells, even between evolutionarily distant species [30]. It remains unclear how epibiotic parasites and predators extract nutrients from the host or prey, however. For *Nanoarchaeum equitans*, electron microscopy showed a close attachment of the parasite to the surface of the host, although no fixed structure was observed [31]. In the case of the bacterial predators *Vampirococcus *and *Ensifer adhearens*, they adhere to the exterior of the prey and appear to attack via a specialized cytoplasmic bridge that is clearly visible as electron-dense materials under the electron microscope [5,32]. The outer membrane of the predator is breached where the dense material appears. Presumably, nutrients can be imported into predators through this junction. It is possible that *Micavibrio *use a similar mechanism to acquire substrates from their prey, as close attachment of *Micavibrio spp*. to prey cells has been shown for strains ARL-13, ARL-14 and EPB previously [6,7,9,11].

### Hemolysin-related proteins

*Micavibrio *grow at the expense of the prey eventually leading to its death. Therefore, it is interesting that *M. aeruginosavorus *encodes six hemolysin-related proteins that belong to the RTX (repeats in the toxin) toxin family, as they all bear the calcium-binding, tandem-repeated GGXGXD signature motif in their sequences (Table 2). RTX toxins are produced by a broad range of bacteria and represent a diverse group of hemolysins, cytolysins, proteases and bactericides. They bind to the host cell membrane and play important roles in bacteria-host interactions [33]. Functions of many RTX toxins have been well studied, among which the alpha-hemolysin from *E. coli *has been best characterized. After secretion, alpha-hemolysin inserts itself into the host cell membrane, forms a transmembrane pore and lyses the cell [34]. It has been suggested that bacteria may use hemolysin to obtain nutrients from the host cells (e.g., irons released from lysed red blood cells) [35].

**Table 2:** Hemolysin-related proteins encoded by *M.aeruginosavorus *

Gene	Length (aa)	No. of Hemolysin-type calcium binding repeat	Other Motifs	Type I secretion system signal	GEI^a ^in attachment/attack phase	Located within a genomic Island
GMV0092	559	6		+	30.0/124.4	
GMV0093	495	0		+	1.8/27.2	
GMV0107	2892	5	Von Willebrand factor	+	16.9/0.3	
GMV0287	1876	11		+	4.1/1.5	+
GMV1777	1296	17		+	4.8/2.2	+
GMV2456	1238	18	Lectin	+	0.4/0.1	+

**^a^GEI: **gene expression index

The hemolysin-related proteins encoded in the *M. aeruginosavorus *genome vary greatly in length and structural features (Table 2). Further examination of their sequences suggests that they might play important roles in prey recognition and adhesion as well. In addition to the glycine-rich tandem-repeats, two proteins also contain motifs known to mediate cell adhesion and recognition. For instance, GMV2456 contains a bacterial lectin-like domain. Numerous bacterial species produce surface lectins, which are calcium-dependent carbohydrate binding modules typically associated with pili. It is well known that bacterial lectins mediate cell-cell recognition and play key roles in infection by promoting bacterial adherence to the host cells [36]. An early study demonstrated that carbohydrate receptors are involved in *Micavibrio*-prey interaction [37], although a recent study suggested this needs to be further investigated [38]. Cell adhesion can be boosted further with two Von Willebrand factor (VWF) type A domains identified in GMV0107. VWF domain mediates cell-cell adhesion via metal ion-dependent adhesion sites [39]. It was originally discovered in extracellular eukaryotic proteins but recently was found to be widespread in bacteria as well.

Notably, hemolysin-related protein is one of few protein families that have been expanded in the *Micavibrio *genome. Phylogenetic analysis indicated that the expansion is not a result of recent gene duplications. In light of the strong genome streamlining in *Micavibrio*, we argue that hemolysin-related proteins play an important role in predation in order for the family to expand and to be maintained in the genome. This is supported by our transcriptomic analysis showing five of the six hemolysin-related genes were actively expressed in either the attack, the attachment, or both stages (Table 2). It is possible that once *M. aeruginosavorus *attaches to a prey cell, it releases hemolysins into the cell junction, which can then insert themselves into the cell membrane of the prey cell, form pores and open up channels for substrates trafficking. The finding that *Bdellovibrio *insert their own outer membrane pore proteins into the prey cell membrane supports this hypothesis [40,41].

### Secretion system and degradative hydrolytic enzymes

The genome of *M. aeruginosavorus *contains a complete type I and a functional type II secretion systems for protein secretion. However, there is no evidence for the presence of type III or IV secretion system. Type I secretion system transports various substances like RTX-toxins, proteases, lipases, and S-layer proteins to the extracellular space, many of which are important in bacteria pathogenesis. The six hemolysin-related genes in *M. aeruginosavorus *genome all possess type I secretion signals and therefore are predicted to be extracellularly translocated by the type I secretion pathway. In *E. coli *and other bacteria, the genes encoding alpha-hemolysin (*hlyA*) and type I secretion system components (*hlyB *and *hlyD*) are transcribed as one operon [42]. Interestingly, GMV0107, the largest hemolysin-protein in *M. aeruginosavorus *genome with 2892 amino acids, is located immediately upstream of a cluster of genes encoding type I secretion system components *TolC *(GMV0108), *hlyB *(GMV0110) and *hlyD *(GMV0111). It has been suggested that this arrangement allows the timely export of toxins without damage to the membrane of the bacteria producing them [42].

Type II is responsible for the extracellular secretion of toxins and hydrolytic enzymes, many of which contribute to pathogenesis in both plants and animals. Proteins secreted through the type II system depend on the Sec or twin-arginine translocation (TAT) system for initial transport into the periplasm. The genome encodes a complete TAT secretion system (*TatABCD*), and a complete Sec secretion system (*SecABDEFGY, YajC, FtsY, SRP*). The type II secretion apparatus is composed of at least 12 different gene products that are thought to form a multiprotein complex. Some components of the type II secretion system, including *GspCGHK*, are absent in the genome annotation. It is possible that they can be substituted by type IV pilus proteins encoded in the genome, as they are homologous and functionally equivalent [43]. Based on the presence of the complete TAT and Sec transport systems, we think the type II secretion system is likely to be functional.

*M. aeruginosavorus *encodes an impressive arsenal of hydrolytic enzymes. A large fraction of the genome (4.3%) was predicted to encode 49 proteases and peptidases, 12 lipases, 2 DNases, 4 RNases and 37 other hydrolases (Additional file 2). Although hydrolytic enzymes are required for the routine maintenance of cellular structures, we expect a sizeable portion of *Micavibrio*'s hydrolytic enzymes to be devoted to digest the prey cell macromolecules. For example, it has been demonstrated that a lytic proteinase of around 39 kDa (+/- 1.5 KDa) isolated from *Micavibrio admirandus *is able to lyse *E. coli *cells [44]. *M. aeruginosavorus *encodes one proteinase in this molecular weight range -- GMV0053 is predicted to encode a 40 kDa peptidase M23 family protein. Although their roles in *Micavibrio *predation remain to be elucidated, with the gene sequences now it is possible to have the hydrolases heterologously expressed and experimentally characterized, as they may be valuable for the development of enzyme-based anti-microbial agents.

### Flagellum and pili

*Micavibrio spp*. are motile and possess a single, sheathless, polar flagellum. Motility gives *Micavibrio *the advantage of being able to actively search for prey. In addition, *M. aeruginosavorus *is capable of biofilm predation [6,8]. Flagellum might provide the necessary force for the predator to penetrate and attack biofilms, as demonstrated in *Bdellovibrio *[45]. As expected, *M. aeruginosavorus *encodes a plethora of genes related to flagellum biosynthesis and chemotaxis (Additional file 3). The genome also possesses multiple dispersed *pil *genes encoding type IV pili, including three operons encoding eight proteins with prepilin-type cleavage/methylation signal at the N-terminus. Proteins with prepilin-like leader sequences are typically involved in type IV pili biogenesis or type II secretion system [46]. Type IV pili in bacteria are in general involved in adherence and invasion of host cells [46] and is believed to play a role in *B. bacteriovorus *predation [47,48]. Although *Micavibrio *are epibiotic predators and do not invade prey cells, type IV pili can play an important role in predation by mediating cell adhesion. This is supported by our transcriptomic data showing that four pili-related genes were highly expressed in the attack or attachment phase (GMV0530, 0902, 0903,1530, see Additional file 4). Notably, gene GMV0530 encoding a *flp/Fap *pilin component family protein was one of the most actively transcribed genes in the attack phase.

### Signal transduction and quorum-sensing

Unlike other obligate parasitic bacteria such as *Mycoplasma *that live exclusively inside the prey cell, *M. aeruginosavorus *is an epibiotic predator constantly exposed to the environment. Moreover, in the attack phase it has to actively search for its next prey. *M. aeruginosavorus *is poised to respond to diverse environmental cues through a suite of signal transduction pathways and processes. For example, the organism has at least 41 genes of two-component signal transduction systems, which is remarkable given its genome size. Intriguingly, the *M. aeruginosavorus *genome encodes at least four genes involved in quorum-sensing: one autoinducer synthase (*LuxI*, GMV1999), two autoinducer binding proteins (*LuxR*, GMV0289 and 0290) and one regulator protein (*LuxO*, GMV1999). Quorum sensing is important for group predation, which requires a quorum of predators and coordinated release of hydrolytic enzymes to degrade the prey. "Wolf pack" predation has been observed in *Myxobacteria *and *Lysobacter *but not in *Micavibrio *or *Bdellovibrio*, at least under laboratory conditions. *Micavibrio *are known to attack the prey on an one-to-one basis [7,9,11], so it is not clear what the biological role of the quorum-sensing genes is. One possibility is that *Micavibrio *can use quorum-sensing to detect their own density and avoid having two or more predators attacking the same prey cell. Multi-predation on a single cell can spell disaster because one prey cell usually does not have enough resource to support the replication of multiple predators. It is also possible that *Micavibrio *can use quorum-sensing to detect the density of the prey population when predating on biofilm. Our RNA-Seq data show that *LuxO *was expressed at low level during the attack phase but not in the attachment phase, *LuxR *was expressed at low level in both phases while *LuxI *was not expressed in either phase (Additional file 4). It will be extremely interesting to elucidate the biological function of the quorum-sensing genes in *Micavibrio*, to investigate whether *Micavibrio *are capable of quorum-sensing, and if so, to deduce its role in the evolution of predation.

### Lateral gene transfers

Since *M. aeruginosavorus *preys on other Gram-negative bacteria, it has the potential to take up prey's DNAs during the feeding process and incorporate them into its own genome. Using BLAST search, we did not find any examples of highly similar stretches of DNA (> 100 bp and 97% identity) shared between *M. aeruginosavorus *and *P. aeruginosa*, the strain that has been used in the laboratory to maintain *Micavibrio*. Similarly, there is no evidence of recent lateral gene transfer from prey into *B. bacteriovorus *[14]. Foreign DNA usually has a nucleotide composition distinct from that of the native DNA and therefore can be detected using chi-square test of base homogeneity, although sequence bias can arise from other sources as well. Our tri-nucleotide chi-square analysis identified numerous regions deviating significantly from the rest of the genome (Figure 1). Among them are operons encoding the rRNA genes and ribosomal proteins, where sequence biases are most likely due to either secondary structure constraint (rRNAs) or biased codon usage (ribosomal proteins). However, we also identify nine genomic islands of possible foreign origins (Additional file 5). Their sizes range from 11.4 Kbp to 27.4 Kbp.

Features found on these islands suggest that they belong to a group of integrative and conjugative elements (ICEs). Four out of nine islands are flanked by tRNA genes on one side and seven out of nine contain the signature integrase related to lambda phages (Additional file 5). tRNA genes are known hotspots for ICE insertion [49,50]. Some also contain helicases, DNA primase, resolvase and reverse transcriptase, mobilization gene (e.g., *mobA/L*) and addiction modules important for ICE maintenance. ICEs normally replicate as part of the host chromosome. But under certain conditions, they can excise from the chromosome, circularize and then transfer to new hosts by conjugation. ICEs therefore combine features of phages and plasmids and can mediate lateral gene flow between distantly related bacterial species [49,50]. It is not immediately clear whether any of the *Micavibrio *ICEs are still functional, i.e., whether they can move within the genome or to other bacterial species. Our transcriptomic data show that at least five integrases were actively expressed during the attachment or attack phase, suggesting that the ICEs can be active.

ICEs allow bacteria to rapidly adapt to new environmental niches [50] and often carry genes such as antibiotic resistance genes and virulence genes (e.g., adhesins, toxins, invasins on the pathogenicity island) [51,52] that confer selective advantages to the cell. *M. aeruginosavorus *strain ARL-13 was originally isolated from sewage water. Not surprisingly, heavy metal (copper, cobalt, zinc, cadmium) resistance genes are found within the *M. aeruginosavorus *genomic islands. Interestingly, three hemolysin genes are also located on the ICEs, in addition to a few genes encoding peptidoglycan binding proteins (Additional file 5).

Since ICEs can move between distantly related species by conjugation, it is natural to ask where did the ICEs in *Micavibrio *come from? ICEs have been found in many bacteria including *Micavibrio*'s prey, *P. aeruginosa*. It is possible, at least in theory, that ICEs are passed from the prey to *Micavibrio *during predation. After all, epibiotic predation and conjugation share an unmistakable common ground -- both involve intimate cell-cell contact and interaction. Phylogenetic analysis of the integrase genes does not support prey being the ICE source. Instead, it indicates that *Micavibrio *ICEs are mostly closely related to those of other alpha-proteobacteria. Therefore, these ICEs either only move among alpha-proteobacteria, or they were present in the ancestor of *Micavibrio *and have been inherited through vertical descent.

### Transcriptome analysis

To identify genes important in the predatory life cycle of *Micavibrio*, we analyzed the transcriptomes of *M. aeruginosavorus *in the attachment and attack phases using RNA sequencing (RNA-Seq). We obtained a total of 8,451,083 reads by Illumina sequencing. 96% of the attack and 60% of the attachment reads were mapped unambiguously to the *M. aeruginosavorus *genome. Of the unmapped reads, the vast majority (92%) were actually the sequences of the prey *P. aeruginosa*. This shows that the prey cells coexisted with the predator cells in the attachment phase but were nearly absent in the attack phase, indicating our strategy of obtaining *Micavibrio *cells at both stages was working. Although we estimated that more than 90% of ribosomal RNAs had been removed during the mRNA preparation, they still constituted the bulk of our illumina reads, as seen previously [53].

Approximately 72.6% of the genome (coding and non-coding) is covered by at least one read, suggesting that more than 27.4% of the genome was not transcribed or was transcribed at low levels in either phase. In addition, 91.6% of reads match predicted ORFs, indicating that there was very little background noise due to potential DNA contamination in our mRNA preparation. RNA-Seq has provided reliable quantitative estimates of gene expression in yeast and bacteria [53-55]. To allow for quantitative comparisons between samples, we calculated the gene expression index (GEI) as the mean coverage depth of the gene normalized by the total number of reads mapped to non-rRNA regions of the genome. Additional file 6 shows a tight correlation between GEI and the transcript level determined by real-time quantitative reverse transcription PCR (qRT-PCR, R^2 ^= 0.85), confirming that our RNA-Seq data provide reliable estimates of gene expression. In addition, as we show below, the expression levels of genes within a particular pathway are fairly consistent, indicating that there was little bias in our RNA-Seq library construction. For example, our RNA-Seq data show strong up-regulation of gene expression in all 54 ribosomal proteins encoded in the genome in the attachment phase.

The transcriptome differs substantially between the attack and attachment phases. Overall, 80.0% of genes were transcribed in the attachment phase, but only 33.4% of genes were transcribed in the attack phase. Genes that were up-regulated in the attack phase are flagellar genes, chemotaxis genes and many hypothetical genes. Genes that were up-regulated in the attachment phase include housekeeping genes involved in DNA replication (e.g., chromosome replication initiation protein, DNA polymerase, DNA topoisomerase, helicase, gyrase), transcription (e.g., RNA polymerase, sigma 70, transcription terminator), translation (e.g., ribosomal proteins, translation initiation and elongation factors), energy production (e.g., TCA cycles, electron transport system, ATP synthase) and cell division (e.g., Fts proteins, cell shape determining factor MreB) (Additional file 4). The gene expression pattern is consistent with what we know about the life cycle of *Micavibrio*. During the attack phase, powered by a single polar flagellum attached at one end of the cell, *Micavibrio *seek out their prey. Once attached to the prey, *Micavibrio *lose their motility, start to feed on their prey, grow, and multiply by binary fission [7,9,11]. Accordingly, genes involved in chemotaxis and flagella biosynthesis were highly expressed in the attack phase but were shut down in the attachment phase. Genes of the two-component signal transduction system were also up-regulated in the attack phase. On the other hand, genes involved in active cell growth and division were highly expressed in the attachment phase, providing the necessary energy and other resources for the cell to replicate. Our genome-wide expression data is consistent with the fact that *M. aeruginosavorus *is an obligate predator that depends on prey to multiply and lacks the ability to propagate in rich media.

Genes involved in protein secretion were also substantially up-regulated in the attachment phase. For example, our RNA-Seq data reveal a uniform increase of gene expression of the entire Sec secretion system (*SecABDEFGY, YajC, FtsY, SRP*), averaging a 17-fold increase when compared to the attack phase. Similarly, the entire twin-arginine translocation (TAT) system, the type I secretion system, and most of the type II secretion system were also significantly up-regulated. This is in agreement with the idea that while attached to the prey cells, *Micavibrio *actively inject hydrolytic enzymes and toxins into prey cells for prey degradation and nutrient uptake. The expression levels of hydrolytic enzymes were nearly unchanged (attachment/attack = 1.29). It is possible that hydrolytic enzymes are produced and accumulate in the attack phase, which can then be readily discharged in the next round of attachment phase.

Interestingly, three cold-shock protein genes (GMV0274, 1414, 2249) were highly expressed in the attachment phase but were not transcribed in the attack phase. Cold-shock proteins of *E. coli *act as mRNA chaperons to promote single-strandedness of mRNA molecules at low temperature to facilitate their translation [56]. A recent study in *Bacillus subtilis *demonstrated that cold-shock proteins are also essential for cellular growth and efficient protein synthesis at optimal growth temperature [57]. Since the attachment cells were never exposed to cold shock before they were mixed with RNAlater, we believe the up-regulation of cold-shock protein genes in *M. aeruginosavorus *serves to maximize the translation efficiency [58]. This is consistent with our observation that genes involved in the translation process were all up-regulated in the attachment phase. Intriguingly, although the heat-shock protein sigma 32 was highly expressed in both phases, its expression was further boosted in the attack phase by 12-fold. Heat shock has been shown to induce axenic growth of *B. bacteriovorus *in rich media, possibly by generating or simulating signals normally derived from prey [59]. Sigma 32 is one of the few functionally characterized genes that were up-regulated in *Micavibrio *during the attack phase, suggesting that it might play an important role in the attack phase by promoting the transcriptions of other genes.

The most highly expressed gene (other than the rRNA genes) in the attachment phase is a porin-encoding gene GMV0043. Porins form aqueous channels on the outer membrane of Gram-negative bacterial cells, and control the diffusion of small metabolites like sugars, ions and amino acids across the outer membrane. GMV0043 was expressed at low level in the attack phase but was dramatically up-regulated in the attachment phase by more than 400-fold. The timing and intensity of the gene expression strongly argue that it plays a critical role in the attachment phase by facilitating the uptake of small metabolites derived from degrading prey cells. Similarly, Lambert et al. have showed that the maltose porin gene in *Bdellovibrio *is highly upregulated during predation, when sugars derived from the prey degradation are available for uptake [60]. Of the five other porin-encoding genes identified in the *Micavibrio *genome, four were actively transcribed in the attachment phase, albeit at subdued levels (GMV0953, 1742, 1033, 0975, see Additional file 4).

Strikingly, most of the highly expressed genes in the attack phase are hypothetical genes. This is in sharp contrast to the gene expression pattern of the attachment phase, where most of the highly expressed genes are well-known housekeeping genes. The fact that the hypothetical genes are highly expressed and the RNA-Seq reads match nicely to the gene models suggest that they are real genes. While uncharacterized, they most likely code for actual proteins that play cryptic but important functions in the unique lifestyle of *Micavibrio*.

## Conclusions

The phylogenomic and transcriptomic analyses of *M. aeruginosavorus *revealed many features consistent with what we know about its epibiotic predatory lifestyle. Analysis of the genome has also provided new perspectives on the biology of this species and the evolution of bacterial predation in general. Because of the lack of good genetic tools for *Micavibrio*, their predation has remained molecularly enigmatic. The analysis reported here and the availability of the complete genome sequence should open up new opportunities and catalyze future studies of this organism.

## Methods

### Bacteria culture and genomic DNA preparation

*M. aeruginosavorus *strain ARL-13 was used in this study [7,8]. *M. aeruginosavorus *was maintained as plaques in double-layered diluted nutrient broth (DNB) agar, a 1:10 dilution of nutrient broth amended with 3 mmol l^-1 ^MgCl_2 _6H_2_O and 2 mmol l^-1 ^CaCl_2 _2H_2_O [pH 7 2] and agar (0 6% agar in the top layer). To initiate a lysate, cocultures were obtained by adding a plug of agar containing *M. aeruginosavorus *plaque to washed overnight grown *P. aeruginosa *PA14 prey cells (1 × 10^9 ^CFU ml^-1^) in DNB and incubated at 30°C on a rotary shaker set at 200 rev min^-1 ^until the coculture became clear (stock lysate). To harvest the predators, cocultures were prepared in which 20 ml of washed *P. aeruginosa *PA14 cells were incubated with 20 ml of stock lysate in 200 ml of DNB and incubated for 48 hrs. Thereafter, the cocultures were passed 10 times through a 0.45-μm Millex pore-size filter (Millipore) to remove residual prey and cell debris. The filtered lysate was spun down for 30 min at 15,000 × g. The supernatant was removed and the pelleted cells were taken for chromosomal DNA extraction using Puregene-Genomic DNA purification kit (Gentra systems) [6].

### Genome sequencing and annotation

The genome was sequenced by 3Kbp paired-end 454 pyrosequencing, in the University of Virginia Department of Biology Genome Core Facility, and was assembled using GS De Novo Assembler (Newbler). The initial Newbler assembly contained 21 contigs in one scaffold. The Phred/Phrap/Consed software package was used for quality assessment in genome assembly. PCR and Sanger sequencing was used to close the gaps between contigs to get the complete genome sequence, which was then annotated by the IGS annotation engine [61]. The complete sequence has been assigned GenBank accession number: CP002382. DNA repeats of at least 50 bp with at least 97% sequence identity were identified using the program Vmatch [62].

### Genome tree construction

Protein sequences of 31 housekeeping genes *(dnaG, frr, infC, nusA, pgk, pyrG, rplA, rplB, rplC, rplD, rplE, rplF, rplK, rplL, rplM, rplN, rplP, rplS, rplT, rpmA, rpoB, rpsB, rpsC, rpsE, rpsI, rpsJ, rpsK, rpsM, rpsS, smpB, tsf) *from genomes of interest were identified, aligned, trimmed and concatenated using the software AMPHORA [63]. The concatenated protein sequence alignment was then used to build a maximum likelihood tree using Phyml [64].

### RNA isolation, library construction, and transcriptome sequencing

To isolate RNA from attachment phase *M. aeruginosavorus *cells, cocultures were prepared as before using *P. aeruginosa *PA14 as the prey. The cocultures were incubated for 8 hrs to allow attachment of the predator to its prey. Thereafter, the cocultures were collected in a 50 ml tube and a fraction containing mainly prey-attached *M. aeruginosavorus *cells was isolated by low speed centrifugation at 4,000 × g for 5 min at room temperature. The pellet was then resuspended in 0.5 ml of RNAlater stabilization solution (Applied Biosystems). For isolating RNA from attack phase *M. aeruginosavorus *cells, the cocultures were incubated for 48 hrs allowing the killing of the prey cells and growth and enrichment of the predator. The clear culture was collected and passed 5 times through a 0.45-μm Millex pore-size filter to remove any residual prey and *M. aeruginosavorus *cells which are still firmly attached to the prey. The filtered lysate was spun down at 4°C for 30 min at 15,000 × g and the pellet containing attack phase *M. aeruginosavorus *was resuspended in RNAlater stabilization solution until RNA extraction.

Total RNA for both attachment and attack samples were isolated from bacteria pellet using RiboPure-Bacteria Kit (Ambion) according to the manufacturer's instructions, with genomic DNA removed using DNase I. RNA was quantified using Quant-iT™ RNA Assay Kit (Invitrogen). 23S and 16S rRNA were removed for mRNA enrichment using MICROBExpress Kit (Ambion). RNA quality analysis using Bioanalyzer (Agilent) indicated that about 90% rRNA was removed. cDNA libraries for Illumina sequencing were then constructed using NEBNext mRNA Sample Prep Master Mix Set 1 (New England Biolabs) following the manufacturer's protocol. Libraries were tagged, amplified by 15 cycles of PCR and sequenced with one lane of Illumina GA IIx 43 cycle single-end sequencing.

### RNA-Seq reads mapping and visualization

FASTX-Toolkit [65] was used to split the pooled reads into separate attachment and attack phase categories, and to eliminate the tag barcodes from the reads. We mapped reads from both attachment and attack sample to the *M. aeruginosavorus *genome using Maq [66], allowing up to 2 mismatches to occur. The gene expression index (GEI) was calculated as the mean coverage depth of the gene, normalized by the total number of reads mapped to non-rRNA regions of the genome. The medium coverage of intergenic regions calculated this way was 0.7. Therefore, based on the RNA-Seq coverage, genes were classified into 4 categories using a schema similar to the one described in [53]: 1) not expressed (coverage < 0.7), 2) low expression (0.7 < = coverage < 10), 3) medium expression (10 < = coverage < 25), 4) high expression (coverage > = 25). The gene expression levels were plotted and visualized in Artemis [67].

### Quantitative real-time PCR

Total RNA for attachment phase sample was reverse transcribed to cDNA using *SuperScript*^® ^II Reverse Transcriptase (Invitrogen). The primer premier 5 software was used to design and select optimum primers for an amplification product of about 350 bp. The quantitative RT-PCR was performed with Fast SYBR-Green master mix (Applied Biosystems) in 7500/7500 Fast Real-Time PCR system. Three replicates were conducted for each gene and the average Ct value was obtained (the cycle number when the fluorescence is detected above the background level). The relative abundance for each gene was calculated based on the 2^-ΔΔCt ^method [68].

## List of abbreviations

ORF: open reading frame; BALOs: *Bdellovibrio *and like organisms (BALOs); GEI: gene expression index; CRISPRs: **C**lustered **R**egularly **I**nterspaced **S**hort **P**alindromic **R**epeats; LPS: lipopolysaccharide; TCA: the tricarboxylic acid; RTX: repeats in the toxin; VWF: Von Willebrand factor; TAT: twin-arginine translocation; ICEs: integrative and conjugative elements; RNA-Seq: RNA sequencing; qRT-PCR: real-time quantitative reverse transcription PCR; T4SSs: type IV secretion systems; CFU: colony forming unit.

## Competing interests

The authors declare that they have no competing interests.

## Authors' contributions

ZW carried out laboratory work of genome and transcriptome sequencing and bioinformatic analysis and contributed to manuscript writing. DK prepared the cell cultures for genome and transcriptome sequencing and helped to draft the manuscript. MW conceived and designed the experiments, analyzed the data and wrote the paper. All authors read and approved the final manuscript.

## Supplementary Material

Additional file 1 **Comparison of major metabolic pathways between *Micavibrio aeruginosavorus*, *Bdellovibrio bacteriovorus *and *Escherichia coli***. A word file listing the number of genes identified in each pathway with the percentage of the genome in parentheses. Click here for file

Additional file 2 **Hydrolytic Enzymes encoded by *M. aeruginosavorus***. A word file listing hydrolytic enzymes identified in *M. aeruginosavorus *genome and their predicted locations by pSort. Click here for file

Additional file 3 **Flagellum biosynthesis and chemotaxis genes of *M. aeruginosavorus***. A word file listing genes involved in flagellum biosynthesis and chemotaxis. Click here for file

Additional file 4 **Gene expression index (GEI) derived from RNA-Seq**. A excel file listing the gene expression index for all ORFs of *M. aeruginosavorus *in the attachment and the attack phases. Click here for file

Additional file 5 **Genomic islands in *M. aeruginosavorus *ARL-13**. A word file listing the genomic islands and their locations, sizes and the genes of interest. Click here for file

Additional file 6 **Correlation between qRT-PCR and RNA-Seq**. An image file in PNG format showing the correlation between qRT-PCR and RNA-Seq data for selected genes in the *Micavibrio *attachment sample. Genes were selected to represent a broad range of gene expression levels. They were: GMV0043 (porin), GMV0092, GM0093, GMV0107 (hemolysin-related proteins), GMV1700 (flagellar hook-basal body complex FliE family), GMV2023 (bacterial regulatory tetR family protein) and GMV2138 (ribosomal protein S7). Click here for file
