# Supplementary material for: Genomic insights into an obligate epibiotic bacterial predator: Micavibrio aeruginosavorus ARL-13
Source: BMC Genomics. 2011 Sep 21;12:453. doi: 10.1186/1471-2164-12-453 (PMC3189940; doi:10.1186/1471-2164-12-453)
Supplement: Additional file 1 — Comparison of major metabolic pathways between Micavibrio aeruginosavorus, Bdellovibrio bacteriovorus and Escherichia coli. A word file listing the number of genes identified in each pathway with the percentage of the genome in parentheses. [file 1471-2164-12-453-S1.DOC]

| Main metabolic features | *M. aeruginosavorus* | *B. bacteriovorus* | *E. coli* |
| --- | --- | --- | --- |
| Amino acid biosynthesis | 37 (1.52%) | 76 (2.09%) | 115 (2.13%) |
| Purines, pyrimidines, nucleosides, and nucleotides | 30 (1.23%) | 76 (2.09%) | 82 (1.52%) |
| Fatty acid and phospholipid metabolism | 32 (1.31%) | 82 (2.26%) | 70 (1.30%) |
| Biosynthesis of cofactors, prosthetic groups, and carriers | 40 (1.64%) | 101 (2.78%) | 104 (1.93%) |
| Central intermediary metabolism | 5 (0.21%) | 145 (4.00%) | 73 (1.35%) |
| Energy metabolism | 99 (4.07%) | 369 (10.17%) | 397 (7.36%) |
| Transport and binding proteins | 89 (3.66%) | 344 (9.48%) | 321 (5.95%) |
| DNA metabolism | 86 (3.53%) | 151 (4.16%) | 107 (1.98%) |
| Transcription | 35 (1.44%) | 73 (2.01%) | 45 (0.83%) |
| Protein synthesis | 117 (4.81%) | 156 (4.30%) | 121 (2.24%) |
| Protein fate | 140 (5.75%) | 230 (6.34%) | 117 (2.17%) |
| Regulatory functions & Signal transduction | 107 (4.40%) | 191 (5.26%) | 175 (3.24%) |
| Cell envelope | 164 (6.74%) | 391 (10.77%) | 180 (3.34%) |
| Cellular processes | 83 (3.41%) | 263 (7.25%) | 190 (3.52%) |
| Mobile and extrachromosomal element functions | 16 (0.66%) | 8 (0.22%) | 50 (0.93%) |
