# Supplementary material for: Genomic insights into an obligate epibiotic bacterial predator: Micavibrio aeruginosavorus ARL-13
Source: BMC Genomics. 2011 Sep 21;12:453. doi: 10.1186/1471-2164-12-453 (PMC3189940; doi:10.1186/1471-2164-12-453)
Supplement: Additional file 2 — Hydrolytic Enzymes encoded by M. aeruginosavorus. A word file listing hydrolytic enzymes identified in M. aeruginosavorus genome and their predicted locations by pSort. [file 1471-2164-12-453-S2.DOC]

| **Categories** | **Predicted location** | **Number** | **Gene** |
| --- | --- | --- | --- |
| **Proteases/Peptidases** |  | Σ49 |  |
|  | Extracellular | 2 | GMV0435, GMV1493 |
|  | Periplasmic | 2 | GMV2106, GMV0400 |
|  | OuterMembrane | 1 | GMV1190 |
|  | CytoplasmicMembrane | 15 | GMV1189, GMV1323, GMV1332, GMV1734, GMV1942, GMV2330, GMV2447, GMV2468, GMV2469, GMV0251, GMV0252, GMV0253, GMV0254, GMV0520, GMV0801 |
|  | Cytoplasmic | 20 | GMV1210, GMV1381, GMV1382, GMV1482, GMV1485, GMV1495, GMV0187, GMV0191, GMV2327, GMV2336, GMV2342, GMV2343, GMV2344, GMV0239, GMV0036, GMV0498, GMV0500, GMV0719, GMV0728, GMV0095 |
|  | Unknown | 9 | GMV1237, GMV1694, GMV1849, GMV2269, GMV0402, GMV0053, GMV0542, GMV0733, GMV0929 |
| **Lipases** |  | Σ12 |  |
|  | Extracellular | 1 | GMV1133 |
|  | CytoplasmicMembrane | 1 | GMV1286 |
|  | Cytoplasmic | 9 | GMV1056, GMV1100, GMV1106,  GMV1602, GMV1603, GMV2060,  GMV2379, GMV0860, GMV0890 |
|  | Unknown | 1 | GMV0881 |
| **Other Hydrolases** |  | Σ37 |  |
|  | Periplasmic | 1 | GMV0451 |
|  | OuterMembrane | 1 | GMV0827 |
|  | CytoplasmicMembrane | 6 | GMV1020, GMV2460, GMV0411,  GMV0425, GMV0448, GMV0737 |
|  | Cytoplasmic | 19 | GMV1024, GMV1309, GMV1318,  GMV1325, GMV1327, GMV1329,  GMV1335, GMV1788, GMV1891,  GMV1913, GMV2181, GMV2396,  GMV0316, GMV0447, GMV0476,  GMV0491, GMV0604, GMV0079,  GMV0934 |
|  | Unknown | 10 | GMV1188, GMV1565, GMV1566,  GMV2004, GMV0318, GMV0450,  GMV0633, GMV0780, GMV0078,  GMV0927 |
| **DNase** |  | Σ2 |  |
|  | Cytoplasmic | 1 | GMV0206 |
|  | Unknown | 1 | GMV0804 |
| **RNase** |  | Σ4 |  |
|  | Cytoplasmic | 3 | GMV1208, GMV1952, GMV0356 |
|  | Unknown | 1 | GMV0812 |
