# Supplementary material for: Genomic insights into an obligate epibiotic bacterial predator: Micavibrio aeruginosavorus ARL-13
Source: BMC Genomics. 2011 Sep 21;12:453. doi: 10.1186/1471-2164-12-453 (PMC3189940; doi:10.1186/1471-2164-12-453)
Supplement: Additional file 3 — Flagellum biosynthesis and chemotaxis genes of M. aeruginosavorus. A word file listing genes involved in flagellum biosynthesis and chemotaxis. [file 1471-2164-12-453-S3.DOC]

| Gene | Description |
| --- | --- |
| **Regulators** |  |
| NA |  |
| **Export apparatus** |  |
| GMV0749 | flagellar biosynthesis protein FlhA |
| GMV1696 | flagellar biosynthetic protein FlhB |
| GMV1697 | flagellar biosynthetic protein FliR |
| GMV1698 | flagellar biosynthetic protein FliQ |
| GMV1707 | flagellar biosynthetic protein FliP |
| GMV0751 | flagellar protein export ATPase FliI |
| **MS-, P- and L-rings** |  |
| GMV1717 | flagellar basal body P-ring formation protein FlgA |
| GMV0767 | flagellar M-ring protein FliF |
| GMV1723 | flagellar P-ring protein (Basal body P-ring protein) |
| GMV1718 | flagellar L-ring family protein |
| **Hook and basal body** |  |
| GMV1716 | flagellar basal-body rod protein FlgG |
| GMV1724 | flagellar FlgJ-like protein |
| GMV2433 | flagellar hook-associated protein FlgK |
| GMV1700 | flagellar hook-basal body complex protein FliE family protein |
| GMV1701 | flagellar basal-body rod protein FlgC |
| GMV1702 | flagellar basal-body rod protein FlgB |
| GMV1727 | flagellar hook capping family protein |
| **Rotor** |  |
| GMV0766 | flagellar motor switch protein FliG |
| GMV0764 | flagellar motor switch protein fliN |
| GMV1713 | flagellar motor switch protein FliM |
| **Motor** |  |
| GMV0763 | motA/TolQ/ExbB proton channel family protein |
| **Filament** |  |
| GMV0775 | flagellin |
| **Chemotaxis** |  |
| GMV2029 | chemotaxis regulator transmitting signal to flagellar motor component |
| GMV2033 | chemotaxis regulator transmitting signal to flagellar motor component |
| GMV1044 | methyl-accepting chemotaxis protein |
| GMV0470 | cheR methyltransferase, all-alpha domain protein |
| GMV0711 | cheR methyltransferase, SAM binding domain protein |
| GMV2027 | cheR methyltransferase, SAM binding domain protein |
| GMV2312 | methyl-accepting chemotaxis (MCP) signaling domain protein |
| **Unknown function within flagellar structure** |  |
| GMV0991 | flagellar basal body-associated protein FliL family protein |
| GMV1714 | flagellar basal body-associated protein FliL family protein |
