# Supplementary material for: Genomic insights into an obligate epibiotic bacterial predator: Micavibrio aeruginosavorus ARL-13
Source: BMC Genomics. 2011 Sep 21;12:453. doi: 10.1186/1471-2164-12-453 (PMC3189940; doi:10.1186/1471-2164-12-453)
Supplement: Additional file 5 — Genomic islands in M. aeruginosavorus ARL-13. A word file listing the genomic islands and their locations, sizes and the genes of interest. [file 1471-2164-12-453-S5.DOC]

| Location | Length (Kbp) | Genes of interest |
| --- | --- | --- |
| GMV0276-0292 | 21.6 | integrase, primase, helicase, hemolysin-related proteins, luxR, tRNA-Thr |
| GMV1004-1026 | 24.8 | polysaccharide biosynthesis, asparagine synthase |
| GMV1243-1260 | 24.2 | integrase, copper resistance gene, mobilization gene, type I restriction enzyme, tRNA-Ser |
| GMV1766-1779 | 15.9 | integrase, hemolysin-related proteins, addiction module, peptidoglycan binding protein |
| GMV1981-2002 | 27.4 | resolvase, helicase, autoinducer luxI, peptidoglycan binding protein, cadmium resistance gene |
| GMV2073-2082 | 13.3 | integrase, primase, reverse transcriptase |
| GMV2168-2176 | 14.9 | integrase, tRNA-Gly |
| GMV2213-2226 | 16.2 | integrase, cadmium resistance gene, mobilization gene, tRNA-Tyr |
| GMV2451-2460 | 11.4 | integrase, hemolysin-related protein, addiction module |
