# Supplementary figures and images for: Genomic insights into an obligate epibiotic bacterial predator: Micavibrio aeruginosavorus ARL-13
Source: BMC Genomics. 2011 Sep 21;12:453. doi: 10.1186/1471-2164-12-453 (PMC3189940; doi:10.1186/1471-2164-12-453)

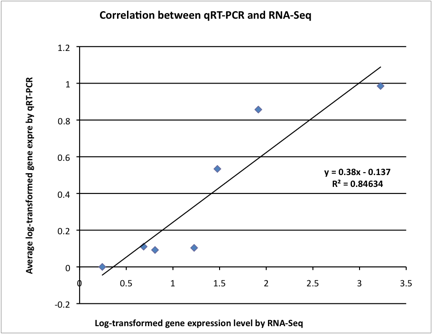

Supplement: Additional file 6 — Correlation between qRT-PCR and RNA-Seq. An image file in PNG format showing the correlation between qRT-PCR and RNA-Seq data for selected genes in the Micavibrio attachment sample. Genes were selected to represent a broad range of gene expression levels. They were: GMV0043 (porin), GMV0092, GM0093, GMV0107 (hemolysin-related proteins), GMV1700 (flagellar hook-basal body complex FliE family), GMV2023 (bacterial regulatory tetR family protein) and GMV2138 (ribosomal protein S7). [file 1471-2164-12-453-S6.PNG]
